# The Demon Alcohol

**Published:** 1896-02

**Authors:** 


### EDITORIAL DEPARTMENT.

F. E. DANIEL, M. D., Editor.
S. E. HUDSON, M. D., Managing Editor.
A. J. SMITH. M. D., Galveston, Associate Editor.

EDITORIAL STAFF:

PROF. J. E. THOMPSON, M. D., Texas Medical College, Galveston; Surgery.
PROF. WM. KEILLER, M. D., Texas Medical College, Galveston; Obstetrics and Gynecology.
PROF. DAVID CERNA, M. D., Texas Medical College, Galveston; Therapeutics.
PROF. A. J. SMITH, M. D., Texas Medical College, Galveston; Medicine.
DR. R. H. L. BIBB, Saltillo, Mexico; Foreign Correspondent.

Official organ of the West Texas Medical Association, the Houston District Medical Association, the Austin District Medical Society, the Galveston County Medical Society, and several others.

### THE DEMON ALCOHOL.

Elsewhere in this issue (Abstracts and Selections) we reproduce a part of a splendid paper by Dr. I. N. Quimby, of Jersey City, read at the Medico-Legal Congress held in New York in September, 1895, and published in the *Medico-Legal Journal* for that month, regretting that we have not space to publish the entire paper. It should set people to thinking.

But, no reform along scientific lines, proposed by medical men, is susceptible of accomplishment without the aid and co-operation of the secular press. Papers of this kind, embodying great truths revealed by science, and having a direct bearing upon the public health and general welfare, and which should be utilized in the framing of our laws, are published only in medical journals and read for the most part only by physicians. For some reason, the daily press persistently refuses to make the public acquainted with them. In Chicago, recently, a Congress of Medico-Legal men was held to discuss needed reforms in criminal jurisprudence, and a number of papers were read, "notable," says the *Journal of the American Medical Association*, "on account of their authorship as well as their contents." The Chicago reporters stated to the officers of the Congress that their respective papers had forbidden them publishing the papers read, and, simply, because they related to prison abuses and other evils, vices amongst prisoners, that need correction, castration in the interests of prophylaxis of crime, etc. Chicago must be becoming a very prude, indeed,—too modest to call a spade a spade; mock modest in refusing to speak of evils existing everywhere, well known to exist; evils which all see and recognize, and to which none can shut their eyes if they would; too "delicate" or squeamish to name the measures science would propose for their cure.

Surely—surely—the time will come when those who are entrusted with the making of the statutes—for the most part representative lay people—merchants, farmers, mechanics, school teachers, with a large sprinkling of lawyers—people who know nothing of the requirements of preventive medicine nor of the sanitary needs of a people, and who are not expected to know—will listen to the dictates of science for reform. The intolerance of legislators in matters of this kind has been repeatedly demonstrated in Texas, when the medical profession, in its organized capacity, and through its most learned and venerable members, has sued in vain for reform in the matter of the regulating the practice of medicine, for instance. They have not only not been heeded, but have been rebuffed with ridicule and derision. Not only in Texas has this spirit been manifested, but in several other States.

There is no more humiliating sight than to witness the utterly disinterested appeals of medical men to the powers, to repeal or modify existing laws and to bring them into accord with the revelations of science, for the better protection of the public health and welfare. Witness the earnest appeal of the San Antonio physicians, to permit them to deal with consumption in the light of modern pathology. (Published in *Texas Medical News* for January.) It is in line, however, with all other efforts of the medical profession to institute prevention—as infinitely better than cure, where cure is even possible—and for the benefit of the public.

But, until Congress is made to see the necessity for it, and creates a Department of Public Health, it can never be hoped that the great evils of intemperance and crime will be intelligently dealt with by legislators. May the day soon come. We commend Dr. Quimby's thoughtful and earnest paper to the serious consideration of our readers. It should be published in pamphlet form and distributed all over the land. So long as the State licenses saloons, so long will the problem of how to lessen crime and criminals remain unsolved. Alcohol is the largest factor in the production of criminals, and causes more sin, suffering, misery, poverty and crime than all other causes combined. The State should make the traffic *odious:* and the habitual use of liquor by any man should bar the doors of society and business alike to him.

It is gratifying to note that the effects of narcotics is to be taught in the text-books of the common schools.

